# Distinct temporal characteristics of circulating alveolar epithelial and endothelial injury markers in ARDS with COVID-19

**DOI:** 10.1186/s13054-021-03596-4

**Published:** 2021-05-17

**Authors:** Kentaro Tojo, Natsuhiro Yamamoto, Takahiro Mihara, Miyo Abe, Takahisa Goto

**Affiliations:** 1 Department of Anesthesiology and Critical Care Medicine, School of Medicine, Yokohama City University, 3-9, Fukuura, Kanazawa-ku, Yokohama, Kanagawa 236-0004 Japan; 2 Department of Health Data Science, Graduate School of Data Science, Yokohama City University, Yokohama, Kanagawa Japan

In the most severe cases, coronavirus disease (COVID-19) leads to acute respiratory distress syndrome (ARDS) that is characterised by alveolar epithelial and endothelial injuries [1]. There are several ARDS biomarkers, which reflect alveolar tissue injuries [2]. The level of circulating soluble form of receptor for advanced glycation end-products (sRAGE) is correlated with type-1 alveolar epithelial injuries [3]. The elevation of angiopoietin-2 (ANG-2) indicates endothelial injury in patients with ARDS [4]. The increase in the circulating surfactant protein concentration indicates alveolar barrier disruption in ARDS cases [5]. Recently, Spadaro et al. reported that COVID-19 ARDS is characterised by increase in the circulating endothelial injury markers [6]. However, the detailed temporal characteristics of these markers remain unclear. In this preliminary study, we investigated the levels of circulating sRAGE, ANG-2, and surfactant protein D (SP-D) in serum samples of patients with COVID-19 with or without ARDS.

Patients who were diagnosed with COVID-19 by real-time polymerase chain reaction and admitted to Yokohama City University Hospital from January to August 2020 were included in this retrospective observational study (Ethics Reference Number: B200700100). Serum concentrations of sRAGE, ANG-2, and SP-D were measured using enzyme-linked immunosorbent assay kits (human RAGE: DY1145; human ANG-2: DY623; human SP-D: DY1920; R&D systems, Minneapolis, MN, USA). We compared the concentrations of these markers in patients with and without ARDS on hospital day 1 or 2. Moreover, we analysed temporal changes in these markers during the first 8 hospital days in those with ARDS. ARDS was diagnosed according to the Berlin Definition.

The data of those with and without ARDS were compared with the Mann–Whitney U test. Temporal changes in the markers were analysed using the Friedman and post-hoc Dunn’s tests. The peak day for each biomarker was observed using the Kruskal–Wallis and post-hoc Dunn’s tests. All statistical analyses were performed using Prism 9.0 software (Graphpad Software, San Diego CA, USA). The level of significance was set at *P* < 0.05.

Eleven and ten patients with and without ARDS, respectively, all with COVID-19, were included. Their characteristics are presented in Table 1. ARDS diagnosis was made on hospital day 1 or 2. The initial serum levels of sRAGE and SP-D were significantly higher in the ARDS than in the non-ARDS group; however, no significant difference was observed in the ANG-2 levels (Table 1).

**Table 1:** Patient characteristics

	Non-ARDS (*n* = 10)	ARDS (*n* = 11)	*P* value
Age, median (IQR), years	57 (30–72)	69 (64–76)	0.2428
Male/female, number	8/2	10/1	0.5865
APACHE2 score, median (IQR)	7 (4.75–9.75)	10 (9.00–14.00)	*0.0204
P/F ratio at admission, median (IQR)	395 (304–454)	155 (108–203)	*< 0.0001
Mechanical ventilation use, number	0	11	*< 0.0001
Laboratory data on admission, median (IQR)			
WBC count, cells/μL	4600 (2525–7925)	7000 (5800–9600)	0.1567
Neutrophil count, cells/μL	2258 (1268–5416)	5628 (4292–7350)	*0.0430
Lymphocyte count, cells/μL	1003 (674–1475)	643 (342–788)	*0.0048
Platelet count, ×10^3^ cells/μL	186 (86–317)	220 (176–287)	0.4262
D-dimer, μg/mL	0.67 (0.00–1.04)	1.35 (0.65–2.52)	*0.0404
CRP, mg/dL	0.98 (0.28–2.06)	14.62 (9.09–16.89)	*< 0.0001
Creatinine, mg/dL	0.75 (0.63–0.87)	0.75 (0.59–0.89)	> 0.9999
Total bilirubin, mg/dL	0.55 (0.40–0.78)	0.60 (0.50–1.00)	0.5661
Alveolar tissue injury marker levels on admission, median (IQR)			
sRAGE, pg/mL	896 (402–1718)	2328 (1404–4982)	*0.0079
ANG-2, pg/mL	334 (65–781)	699 (405–2201)	0.1321
SP-D, pg/mL	2407 (772–3833)	16,596 (6733–21,397)	*0.0062

*ARDS* acute respiratory distress syndrome, *ANG-2* angiopoietin-2, *SP-D* surfactant protein D, *sRAGE* soluble form of receptor for advanced glycation end-products, *WBC* while blood cell, *CRP* c-reactive protein, *IQR* interquartile range

Analysis of temporal changes in these markers in 10 patients with ARDS, after excluding one patient with missing data, revealed that the serum sRAGE level peaked just after admission, and gradually decreased with hospital days (Fig. 1). Conversely, serum ANG-2 and SP-D levels did not significantly decrease during the first 8 hospital days and the peak timings of these markers were observed during a later disease stage (Fig. 1).

**Fig. 1:**
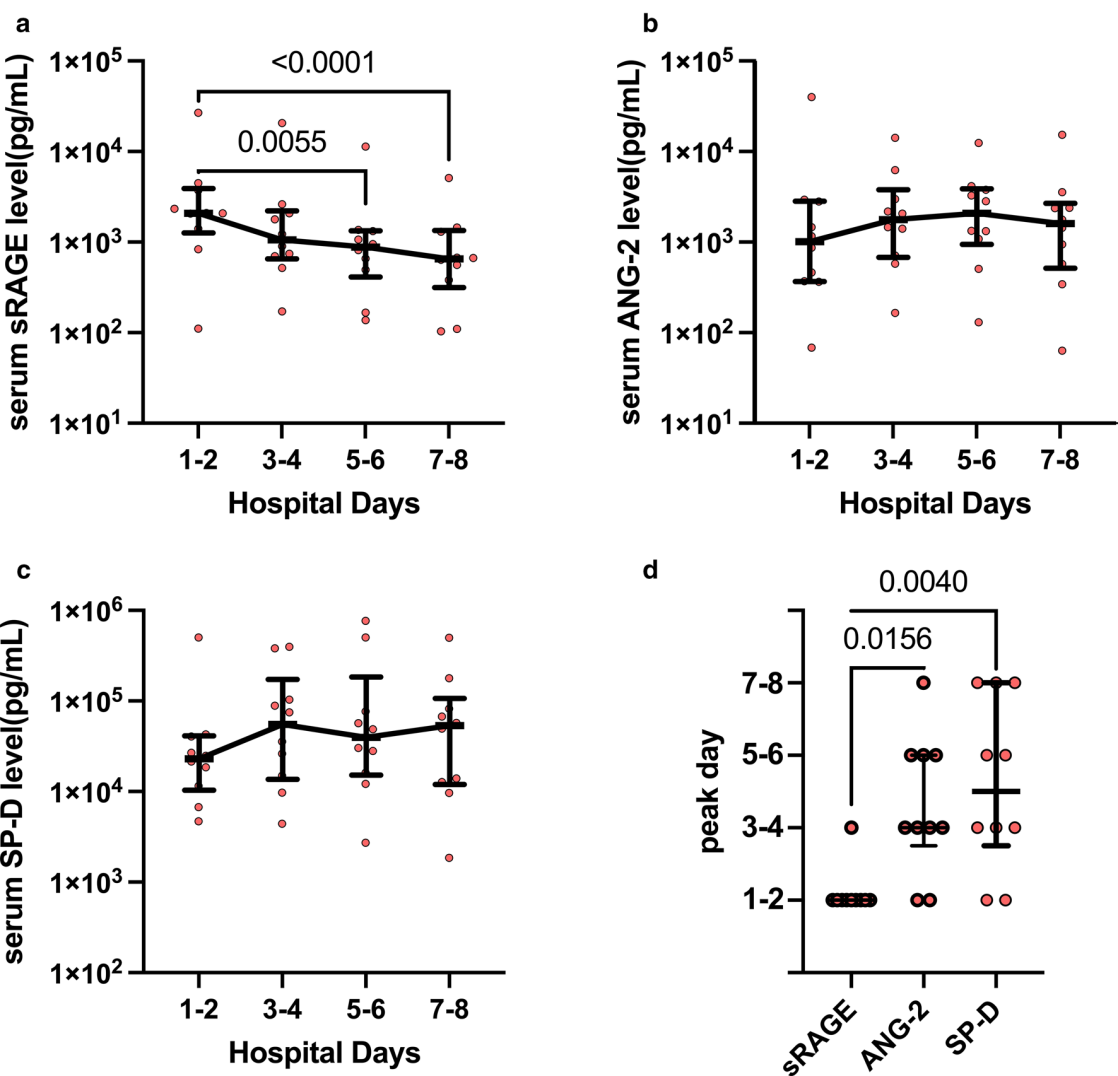
Temporal changes in **a** sRAGE, **b** ANG-2, and **c** SP-D levels. The examination was performed in patients with ARDS with COVID-19 during the first 8 days from admission. In cases where the values for every 2-days were available, the mean values were used; where only a single-day value was available, this value was used. **d** The peak day of each alveolar tissue injury marker is presented. Data are presented as medians ± IQRs. *ARDS* acute respiratory distress syndrome, *ANG-2* angiopoietin-2, *SP-D* surfactant protein D, *sRAGE* soluble form of receptor for advanced glycation end-products

We showed that alveolar epithelial injury occurring at the very early disease stage, indicated by the increased sRAGE level, is a hallmark of COVID-19 ARDS. Conversely, the ANG-2 and SP-D levels peaked at later time points, suggesting that the endothelial injury and alveolar barrier disruption continued to exacerbate for several days after admission.

A study limitation was that the precise mechanism of sRAGE or ANG-2 release from the alveolar epithelial or endothelial cells remains unknown. However, the difference in the peak timing of these markers suggested distinct mechanisms for injury to each cell type. Additionally, it is possible that the initial alveolar epithelial injury might be a trigger of the subsequent exacerbation. Further investigations analysing temporal associations between these markers and inflammatory mediators could help identify the mechanisms underlying alveolar tissue injury. Moreover, the trajectory analysis of these markers linking clinical outcomes could help understand the detailed COVID-19 pathogenesis.

## Data Availability

The datasets used and/or analysed during the current study are available from the corresponding author on reasonable request.

## Abbreviations

ARDS: acute respiratory distress syndrome
COVID-19: coronavirus disease
sRAGE: soluble receptor for advanced glycation end-products
ANG-2: angiopoietin-2
SP-D: surfactant protein D
SARS-CoV-2: severe acute respiratory syndrome coronavirus-2
